# Multiple sclerosis and the limits of classical autoimmune theory

**DOI:** 10.3389/fimmu.2026.1849080

**Published:** 2026-06-18

**Authors:** Anna Karin Hedström, Fredrik Piehl

**Affiliations:** Department of Clinical Neuroscience, Karolinska Institute, Stockholm, Sweden

**Keywords:** autoimmunity, biomarkers, causal inference, disease mechanisms, disease progression, inflammation, multiple sclerosis, neurodegeneration

## Abstract

Concepts of autoimmune disease have traditionally emphasized genetic susceptibility, antigen-specific immune activation, and inflammation as linear drivers of tissue injury and clinical symptoms. While this framework has been highly successful in explaining disease initiation and guiding the development of immunomodulatory therapies, accumulating evidence indicates important limitations in its capacity to account for long-term disease outcomes. In this review, we use multiple sclerosis (MS) as an illustrative case through which autoimmune theory can be critically examined. MS provides strong support for immune-mediated mechanisms in early disease, yet it also shows that neurodegeneration and disability accumulation often become uncoupled from overt inflammatory activity. These observations challenge linear models and sustained coupling between immune activity, tissue injury, and clinical progression. We use this gap between theoretical expectations and observed disease patterns to examine broader conceptual issues relevant to autoimmune and immune-mediated diseases, particularly the need to distinguish between mechanisms of susceptibility, inflammatory activity, and progressive worsening, and to incorporate temporal dynamics, interactions, and tissue-specific responses into explanatory models. We argue that the main contribution of MS lies not in serving as a generalizable model, but in clarifying the scope and limits of autoimmune theory and in underscoring the importance of theoretical refinement for organizing increasingly complex empirical evidence.

## Introduction

### Classical autoimmune theory and its assumptions

Concepts of autoimmune disease have historically been shaped by conditions in which antigen-specific immune responses against self-components are understood to directly produce tissue injury and sustained inflammation, with these processes corresponding closely to clinical manifestations (1–3). Within this framework, disease causation is typically described as a linear sequence in which host susceptibility predisposes to dysregulated immune activation directed against self-antigens, resulting in tissue damage and clinical disease. This conceptual model has been particularly successful in explaining disease initiation and has provided a coherent basis for the development of immunomodulatory therapies across a broad range of autoimmune and immune-mediated disorders (4, 5).

At the same time, this perspective rests on a set of assumptions regarding causality, temporal structure, and disease evolution (1–3). Immune activation is treated not only as the initiating event but also as the principal driver of pathology throughout the disease course. Host susceptibility is implicitly regarded as a stable background condition rather than as a factor whose relevance may vary over time. Inflammation, in turn, serves as the primary indicator of disease activity across disease stages. Together, these assumptions define a model in which immune activity, tissue injury, and clinical manifestations are expected to remain closely coupled over time.

Multiple sclerosis (MS) occupies a distinctive position with respect to these expectations. As an immune-mediated disease of the central nervous system (CNS), MS has long been associated with inflammatory tissue lesions, genetic associations within the major histocompatibility complex, experimental models of transferable autoimmunity, and therapeutic responsiveness to immunomodulatory treatment (6, 7). Collectively, these observations strongly support immune-mediated mechanisms in disease initiation and place MS firmly within the conceptual boundaries of autoimmunity.

However, several key features of MS are not fully accommodated within this view. Despite strong immunological evidence for immune involvement in disease initiation, immune mechanisms do not fully account for the marked clinical heterogeneity, the considerable dissociation between inflammation and disability, or the progressive neurological decline that characterize later stages of the disease (8). These features indicate that immune-mediated inflammation, while central to early disease, is insufficient to explain the full course of the disease.

We propose that MS exposes important limitations of classical linear autoimmune models when these are applied across the entire disease course. Specifically, we argue that the mechanisms underlying disease susceptibility, inflammatory activity, and progressive neurological worsening are mechanistically related but become progressively uncoupled over time. While immune-mediated inflammation plays a central role in disease initiation and early clinical activity, later progression increasingly reflects interactions between compartmentalized inflammation, CNS-intrinsic tissue responses, cumulative injury, and impaired repair capacity. This perspective suggests that MS is better understood as a temporally dynamic and multidimensional disease process rather than as a uniform autoimmune cascade operating across all disease stages.

Using MS as an illustrative case, we examine how immune-mediated inflammation, tissue-specific responses, and neurodegeneration interact across different stages of disease. This approach allows us to identify where prevailing autoimmune models remain explanatory and where additional conceptual integration is needed to account for long-term disease evolution.

## Where autoimmune theory succeeds

### Immune-mediated mechanisms in early MS

Early models of MS were built on observations that aligned closely with prevailing concepts of autoimmune disease. Neuropathological studies consistently demonstrated multifocal inflammatory lesions within the CNS, characterized by demyelination, perivascular immune-cell infiltrates, and relative axonal preservation in early disease stages (9). The close correspondence between inflammation, tissue injury, and clinical manifestations supported the interpretation of immune activity as a primary driver of lesion formation (6, 10). In this context, MS appeared to fit well within an autoimmune framework in which antigen-directed immune responses cause localized tissue damage.

Within this framework, T lymphocytes were identified as key mediators of disease. Early models emphasized CD4^+^ T helper cell-mediated inflammation, including Th1 and later Th17 responses, as contributors to CNS inflammation and demyelination (11–13). These findings expanded rather than displaced earlier immune-centered interpretations, reinforcing the view that multiple effector pathways could give rise to a shared pathological phenotype.

The development of experimental autoimmune encephalomyelitis (EAE) further strengthened this interpretation. EAE demonstrated that inflammatory demyelinating disease of the CNS could be induced through immunization with myelin-associated antigens and, critically, that disease could be adoptively transferred by immune cells (14, 15). This provided direct experimental support for the idea that adaptive immune responses are sufficient to initiate CNS pathology (15, 16). Although EAE was never intended as a complete model of MS, it reinforced confidence in this causal link (16–18).

Antigen specificity was a core assumption of early immune-centered disease models. In EAE, disease induction depended on immunization with defined CNS antigens, establishing a direct link between immune recognition and tissue targeting (14, 15). In MS, the corresponding target antigens remained uncertain, but prevailing interpretations nevertheless assumed that immune responses were directed against CNS components (6, 8, 19). This assumption shaped decades of research aimed at identifying candidate autoantigens and characterizing antigen-driven responses in both central and peripheral compartments.

Prevailing views of the CNS as an immunologically restricted environment further supported this early autoimmune paradigm (20, 21). Limited immune surveillance, the presence of the blood-brain barrier, and the absence of conventional lymphatic drainage were thought to constrain immune access to neural tissue (21–23). Against this background, MS appeared to represent a condition in which these constraints were breached, allowing immune-cell entry and sustained inflammation (24, 25). This framing emphasized immune trafficking and barrier integrity as key elements in disease initiation.

Genetic findings were readily accommodated within immune-centered explanations. Associations within the major histocompatibility complex, particularly HLA class II loci, aligned naturally with mechanisms involving antigen presentation and T cell activation. Although these associations did not identify specific pathogenic antigens or delineate precise disease mechanisms, they were consistent with the view that variation in antigen recognition and immune regulation contributes to disease susceptibility (26–28). Together with pathological and experimental evidence, genetic findings strengthened the view of MS as an immune-mediated disorder driven primarily by adaptive immune dysfunction.

Early clinical responses to anti-inflammatory and immunomodulatory therapies provided important empirical support for immune mechanisms in MS. Treatments that reduced immune activation, lymphocyte trafficking, or inflammatory lesion formation were consistently associated with reduced relapse rates and suppressed radiological disease activity (8, 10, 29). These therapeutic effects demonstrated that targeting these pathways could substantially modify key aspects of disease activity, particularly during early and relapsing phases. In this respect, MS closely aligned with expectations from classical autoimmune models, in which modulation of immune activity leads to measurable clinical benefit.

Taken together, these lines of evidence established a coherent explanatory basis for MS, particularly for disease initiation and early inflammatory activity. Pathological observations, experimental models, genetic associations, and therapeutic responses all supported the interpretation that immune processes play a central role in how disease begins and presents clinically. However, this model proved less able to account for disease evolution beyond early inflammatory stages.

## Genetic susceptibility without determinism

### Scope of genetic explanations in MS

From the earliest genetic and familial studies, MS was recognized as having a substantial heritable component. Twin studies have estimated the heritability of MS to be approximately 50%, with monozygotic twin concordance rates around 25-30% compared with 2-5% in dizygotic twins (30). At the same time, the incomplete concordance among monozygotic twins and the relatively low absolute risk among relatives were consistent with longstanding clinical and epidemiological observations that MS is not a Mendelian disorder and that genetic predisposition alone cannot account for disease occurrence (27, 28, 30). From the outset, MS was therefore understood as a condition in which inherited susceptibility operates alongside non-genetic influences.

The first major genetic associations in MS were identified within the major histocompatibility complex (31). Associations involving HLA class II loci, particularly HLA-DRB1*15:01, implicated antigen presentation and adaptive immune activation (27, 28, 31). These findings supported the role of variation in immune recognition and regulation in disease susceptibility.

As genome-wide approaches expanded, the broader genetic architecture of MS became increasingly clear (27, 28, 32, 33). Genetic risk was found to be distributed across many variants, most with modest effect sizes and many mapping to immune-related genes and regulatory elements (32, 33). These variants are enriched in genes involved in host defense and immune regulation, consistent with the idea that genetic susceptibility to autoimmunity may partly reflect evolutionary trade-offs between protection against infection and the risk of immune-mediated disease. Rather than implicating a small number of high-impact variants, this pattern points to a cumulative and distributed genetic contribution to disease risk.

Genetic studies also clarified the limits of what inherited variation can explain. While genetics has been highly informative with respect to susceptibility, it has been less informative for clinically central features, including the heterogeneity of disease course and the dissociation between inflammatory activity and disability accumulation.

## Infection and temporal structure in MS: the case of EBV

### From triggers to necessary components

Environmental influences and infectious exposures have long been considered in theories of MS etiology. Early epidemiological observations, including geographic gradients, migration effects, and age-dependent risk, suggested that non-genetic exposures play an important role in shaping disease susceptibility (34, 35). In this context, infections were typically conceptualized as potential triggers of disease in genetically susceptible individuals (36, 37), rather than as necessary components of disease risk.

Over time, however, evidence implicating Epstein-Barr virus (EBV) accumulated to a degree that challenged this framing (38–42). Near-universal EBV seropositivity among individuals with MS, elevated antibody titers preceding clinical onset, and longitudinal studies showed that MS rarely develops in the absence of prior EBV infection collectively shifted how infection is positioned in MS causation (38–41). Rather than functioning merely as a non-specific trigger, EBV increasingly appeared to represent a necessary, though clearly not sufficient, component of disease risk. EBV infection is ubiquitous, whereas MS remains rare, indicating that additional causal factors are required for disease to occur.

Recognition of EBV as a necessary component of MS risk reframed infection as a long-term immunological influence rather than an isolated event. EBV establishes lifelong latency within the B cell compartment, shaping immune memory, B cell differentiation, and host-virus equilibrium over extended periods (39–41). EBV infection thus represents a persistent biological influence on the immune system that precedes clinical disease by several years (39, 42).

B cells therefore occupy a central position in this reframing. EBV’s tropism for B cells, together with neuropathological evidence of clonally expanded B cell populations and ectopic lymphoid structures within the CNS, provides a mechanistic link between latent infection and sustained immune activity (41, 43, 44). The clinical efficacy of B cell-depleting therapies further supports the plausibility that B cell-mediated processes contribute not only to disease initiation but also to its persistence (45–47).

How EBV contributes to MS remains an active area of investigation. EBV may initiate autoimmune responses that later become self-sustaining (48, 49), contribute to ongoing inflammation through chronic infection of the B cell compartment (48, 49), or sustain immune activity through EBV-infected B cells within the CNS (50, 51). These mechanisms are not mutually exclusive and may operate in parallel across different disease stages or patient subgroups (38).

The growing evidence linking EBV to MS has not replaced earlier autoimmune models but has expanded them by highlighting how host-pathogen interactions can leave durable imprints on the immune system. The focus has therefore shifted from asking whether infection triggers autoimmunity to examining how prior immune events shape the conditions under which pathogenic immune responses are later generated and maintained (39–41).

Other environmental and lifestyle-related factors, including lung-irritating exposures such as smoking, adolescent obesity, sunlight exposure, and vitamin D-related pathways, have also been associated with both MS susceptibility (52–55) and disease progression (56–58). Several of these factors interact with EBV infection and genetic susceptibility (42, 59, 60), supporting the view that the development and long-term evolution of MS reflect cumulative and context-dependent interactions between infectious, environmental, immunological, and host-related processes. However, while these exposures clearly influence risk and disease course, they primarily act as modifiers within an established susceptibility context, rather than challenging the underlying causal structure of autoimmunity in the same way as EBV.

## Progression beyond inflammation

### Where autoimmune theory reaches its limits

From early pathological descriptions onward, MS was understood to involve irreversible tissue damage and neurodegeneration (9, 10). Axonal loss, gliosis, and permanent neurological impairment were recognized as characteristic features of the disease and major contributors to long-term disability (61, 62). A central question has been to what extent these degenerative processes are driven by immune-mediated inflammation, and whether sufficiently effective suppression of inflammatory activity could prevent or substantially delay irreversible tissue loss (63).

Within autoimmune frameworks, a common assumption has been that inflammatory activity acts as a key upstream driver of neurodegeneration (62, 63). In this view, focal immune-mediated inflammatory lesions initiate tissue injury, and repeated or sustained inflammatory activity over time culminates in axonal loss and clinical progression (10, 62). This view is consistent with both pathological observations and established concepts of autoimmune disease, in which immune attack precedes and mechanistically explains downstream tissue damage.

The success of anti-inflammatory therapies in MS initially appeared to confirm central expectations of autoimmune theory. Suppression of relapse activity and reduction of new inflammatory lesion formation demonstrated that immune processes play a decisive role in early disease activity (64, 65). In relapsing disease, targeting immune pathways translated into clear clinical benefit (65), reinforcing the view that inflammatory activity and disease progression were closely linked, particularly early in the disease course.

**Figure 1:**
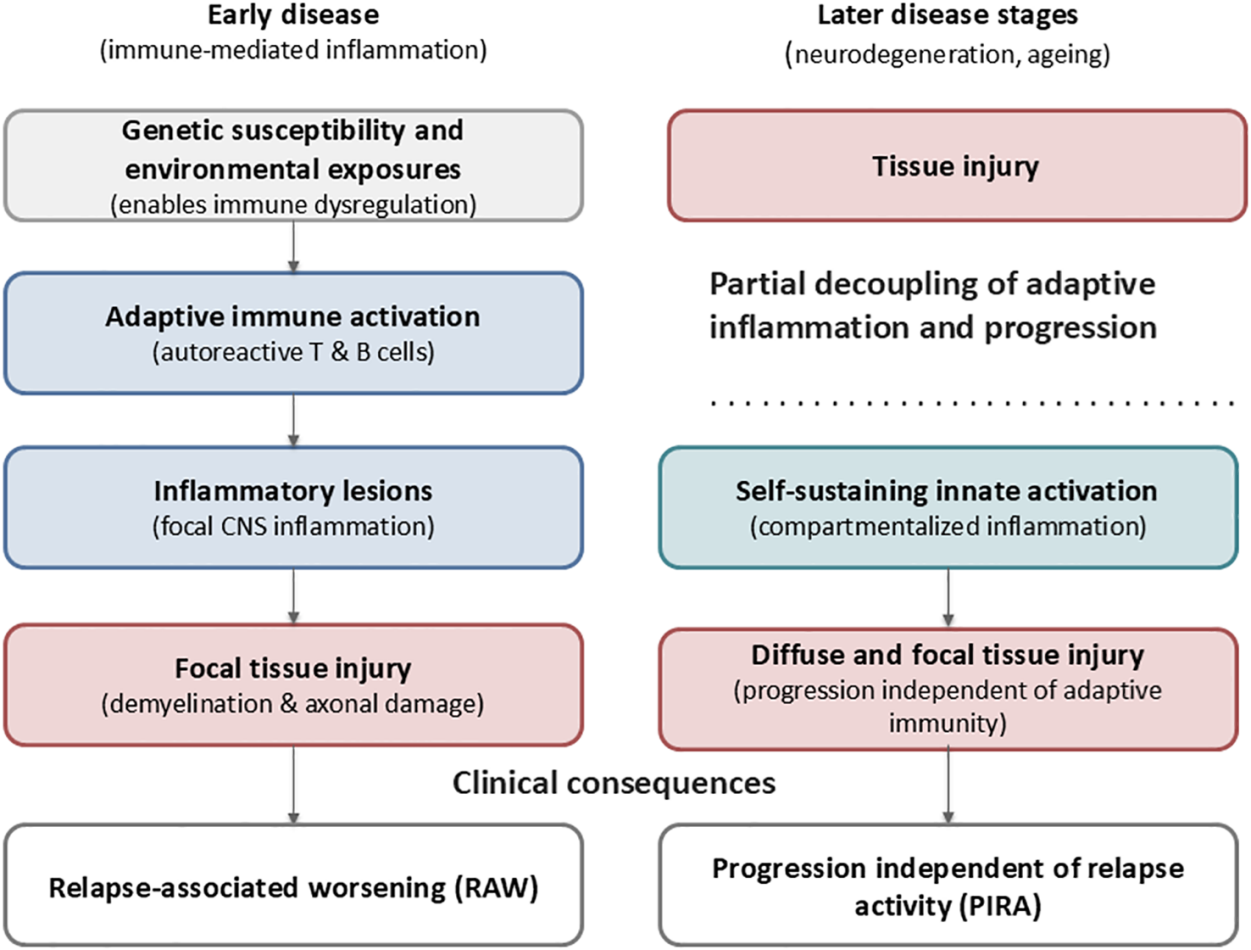
Conceptual model of immune-mediated inflammation and neurodegeneration in multiple sclerosis. In early disease, genetic susceptibility and environmental exposures promote immune dysregulation, leading to activation of adaptive immune responses and the formation of focal inflammatory lesions in the central nervous system. These processes result in tissue injury characterized by demyelination and axonal damage, clinically reflected as relapse-associated worsening (RAW). In parallel, neurodegeneration is associated with self-sustaining innate immune activation, reflecting compartmentalized inflammation that can drive further tissue injury independently of adaptive immunity. This process is clinically expressed as progression independent of relapse activity (PIRA). The image illustrates a partial decoupling of inflammation and progression, whereby neurodegenerative processes become increasingly dissociated from focal inflammatory activity.

At the same time, longitudinal clinical observations revealed a more complex reality. Despite effective control of relapses and conventional markers of inflammatory activity, many individuals continued to experience gradual neurological worsening. Disability accumulation was observed even in the absence of overt inflammatory events, a phenomenon now described as progression independent of relapse activity (PIRA) (66–69). This pattern, illustrated in **Figure 1**, indicates that although inflammation and neurodegeneration are mechanistically linked, suppression of peripheral inflammatory activity alone is often insufficient to prevent progressive tissue loss.

These observations shifted focus toward the location and nature of disease-driving processes. One line of interpretation emphasized compartmentalized inflammation within the CNS. Inflammatory activity was increasingly recognized as persisting behind an intact or partially restored blood-brain barrier, involving meningeal immune aggregates, chronically activated microglia, and locally sustained immune signaling (70–72). This interpretation is supported by the observation that several highly effective therapies, particularly monoclonal antibodies targeting peripheral immune pathways, have limited penetration into the CNS, leaving compartmentalized inflammatory processes relatively less affected (73).

In parallel, increasing emphasis has been given to CNS-intrinsic mechanisms of neurodegeneration. Glial activation, mitochondrial dysfunction, oxidative stress, impaired axonal energy metabolism, and limitations in remyelination and repair have all been implicated as contributors to progressive tissue damage (9, 73). These processes are not easily incorporated into classical autoimmune models centered on autoreactive lymphocytes and antigen-driven responses. Instead, they suggest that as disease evolves, progression becomes increasingly shaped by local tissue responses and cumulative injury rather than by focal immune attack alone.

Several mechanisms implicated in disease progression in MS overlap with processes observed in other neurodegenerative disorders (62, 74). However, unlike primary neurodegenerative diseases, these processes in MS arise within a disease context shaped by prior and ongoing immune-mediated tissue injury. This distinction further supports the view that progression in MS cannot be fully understood through classical models of peripheral autoimmune inflammation alone. Together, the dissociation between inflammatory activity and disability progression, the persistence of compartmentalized CNS inflammation, and the emergence of CNS-intrinsic neurodegenerative processes delineate the limits of autoimmune theory as a comprehensive explanation of disease course. In MS, disease progression can shift from an inflammation-dominated process to one more strongly shaped by intrinsic tissue pathology.

## What MS reveals about autoimmune theory

### From linear causation to integrated models

A recurring theme in MS research has been the mismatch between empirical observations and the assumptions of classical autoimmune theory. Early autoimmune models were predominantly linear, positing a sequence in which genetic susceptibility predisposes to immune dysregulation, immune activation targets self-tissue, inflammation causes damage, and cumulative injury gives rise to clinical manifestations (1–3). This account proved highly effective in explaining disease initiation and in guiding the development of therapies aimed at suppressing immune activation (29). However, as evidence accumulated over longer time scales and across different phases of MS, the limitations of such linear models became increasingly apparent (**Figure 1**).

A key limitation is that linear models implicitly assume continuity between cause and consequence across the disease course. The processes that initiate pathology are expected to remain the primary drivers of disease activity and outcome. MS has repeatedly challenged this expectation. Immune-mediated inflammation is clearly central to disease onset and early lesion formation, yet it does not reliably predict disability accumulation or neurodegeneration (8, 62). This divergence highlights a gap between mechanisms of disease initiation and those underlying persistence or progression.

Rather than invalidating autoimmune theory, these observations underscore the need to distinguish between different dimensions of disease. MS made it increasingly clear that risk, disease activity, and disease progression are related but not interchangeable processes. Genetic and environmental factors shape susceptibility and influence whether disease emerges at all. Inflammatory immune activity largely determines relapses, focal lesion formation, and short-term clinical fluctuations. Progressive tissue loss and functional decline, however, may proceed through additional mechanisms that are only partly dependent on ongoing inflammatory activity (8, 62, 75). Linear models struggle to accommodate these distinctions because they implicitly collapse these dimensions into a single causal chain.

**Figure 2:**
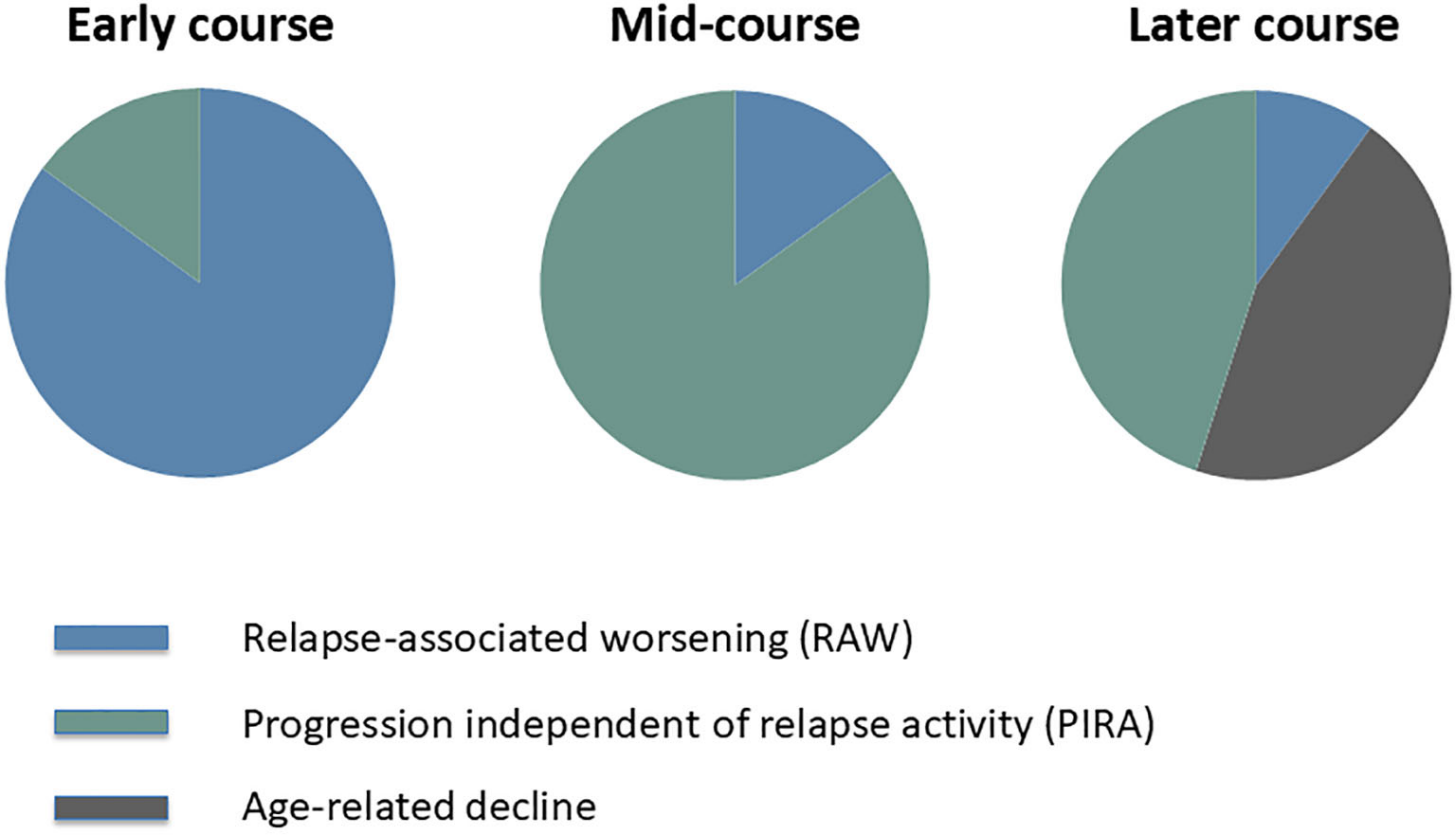
Conceptual representation of disease mechanisms across the course of multiple sclerosis. Across the disease course, the relative contributions of relapse-associated worsening (RAW), progression independent of relapse activity (PIRA), and age-related decline shift. Early disease is dominated by RAW, reflecting inflammatory activity, whereas PIRA becomes increasingly prominent during mid-course disease. In later stages, age-related processes and accumulated tissue damage contribute substantially to disability progression. The image illustrates how different mechanisms of worsening coexist but vary in relative importance over time.

A related insight concerns the temporal organization of disease mechanisms. MS illustrates that different biological processes may dominate at different stages of the disease course without implying discrete phase transitions or mutually exclusive mechanisms (**Figure 2**). Inflammatory activity, tissue injury, neurodegeneration, and limitations in repair capacity are present throughout the disease, but their relative contributions change over time. Early disease is often characterized by prominent inflammatory activity and clinical relapses, whereas later stages are increasingly shaped by diffuse tissue damage, compartmentalized inflammation, and limited repair capacity. This shifting balance of mechanisms is difficult to reconcile with static models that assign a single dominant cause to the disease as a whole (29, 62, 74).

MS therefore reveals a structural limitation within autoimmune theory. It illustrates that autoimmune models must accommodate diseases in which the relationships between disease risk, clinical activity, and long-term outcome are only partly aligned. Rather than abandoning immune causation, such models place it within a broader conceptual framework that incorporates timing, interaction, and context. In this sense, MS serves less as a generalizable model than as a critical stress test, revealing where simplified causal assumptions lose explanatory power.

## Implications for autoimmune disease research

The conceptual developments arising from MS research raise broader questions about how autoimmune diseases are defined, classified, and investigated. In many autoimmune disorders, causal reasoning is centered on a primary immune abnormality, often framed as autoreactive lymphocytes targeting specific self-antigens. While this approach has been highly productive, it can fail to capture the complex interplay between genetic susceptibility, environmental exposures, tissue vulnerability, and temporal dynamics that together influence disease initiation and progression.

### Disease definitions and classification

Traditional definitions of autoimmune diseases emphasize the presence of antigen-specific immune responses, characteristic inflammatory pathology, or responsiveness to immunomodulatory treatment. Such criteria are well suited to identifying immune-mediated disease initiation but may be less informative for later stages of disease, where inflammatory mechanisms may persist alongside additional processes that shape long-term outcomes.

MS illustrates this limitation clearly. Immune-mediated inflammation is central to early disease and provides a strong basis for classification as an autoimmune condition. However, progressive neurological decline may occur with limited correspondence to overt inflammatory activity. Similar patterns have been described in other chronic inflammatory diseases, such as rheumatoid arthritis, where early inflammatory processes can initiate tissue responses that later evolve with only partial dependence on ongoing inflammation (76).

This raises questions of whether autoimmune diseases should be defined primarily by initiating mechanisms, by dominant pathological features at a given time, or by the processes that ultimately determine irreversible tissue damage and disability. The MS example suggests that a single disease label may encompass multiple biological states with partially overlapping but distinct mechanisms.

### Biomarkers and disease monitoring

The role of biomarkers is closely related to disease definition. In MS, markers of inflammatory activity, such as MRI measures of new lesion formation, are highly valuable for assessing short-term disease activity and treatment response. At the same time, their limited value for predicting long-term progression has underscored the need for biomarkers that reflect additional processes, including neuroaxonal injury and glial pathology, as captured by circulating markers such as serum neurofilament light chain and glial fibrillary acidic protein (77, 78).

This pattern extends beyond MS. In autoimmune and immune-mediated diseases, reliance on biomarkers tightly linked to immune activation may preferentially capture reversible or early disease processes while providing limited information about mechanisms that drive cumulative tissue damage. The MS experience illustrates that inflammatory markers can be necessary but not sufficient for assessing cumulative tissue damage and long-term outcomes, highlighting the value of explicitly matching biomarkers to the disease dimension under study.

### Prevention and risk reduction

MS is distinctive in discussions of autoimmune disease prevention because it is one of the few conditions for which a necessary causal factor has been identified. It therefore differs from most autoimmune diseases, where no single necessary exposure has been identified for disease to occur.

The broader conceptual lesson, however, lies in the structure of causation rather than in the specific exposure involved. MS demonstrates that autoimmune risk can be organized hierarchically, with certain exposures acting as gatekeeping conditions for disease, while genetic and environmental factors modulate risk only within that context.

Where such necessary components cannot be identified or eliminated, prevention in the strict sense may not be achievable. In these settings, risk reduction must instead focus on modifying cumulative and interacting influences that shape disease probability across the life course. MS thus clarifies how different causal roles (necessary, modifying, and downstream) can coexist within autoimmune disease etiology.

### Treatment and phase-specific intervention

The most direct implications concern treatment strategies. In MS, therapies targeting B cells or lymphocyte trafficking are highly effective in suppressing inflammatory disease activity early in the disease course, yet their impact on later progression is more limited (79). Recent therapeutic developments further illustrate this distinction. Bruton’s tyrosine kinase inhibitors such as tolebrutinib show relatively modest effects on conventional markers of inflammatory activity, such as MRI lesion formation and relapse frequency, yet may influence the risk of disability progression in progressive disease (80, 81). This pattern suggests that therapeutic effects depend not only on the mechanisms targeted but also on the timing of intervention within the disease course.

The broader implications of these observations extend beyond MS. Similar patterns can be seen in at least some chronic inflammatory diseases, such as rheumatoid arthritis, in which early inflammatory mechanisms initiate tissue injury, whereas later stages are increasingly shaped by interactions between immune activity, local tissue responses, and repair processes (82, 83). Such dynamics challenge explanations that attribute disease expression to a single dominant mechanism and instead support approaches that incorporate temporal variation in disease mechanisms.

### Broader relevance and limits of generalization

Taken together, the preceding arguments indicate that MS serves as a valuable case for autoimmune disease research, not as a universal model, but as a case in which key conceptual limitations become particularly visible. The need to distinguish between disease dimensions, to align biomarkers with underlying biological processes, and to adapt preventive and therapeutic strategies to disease phase is unlikely to be unique to MS, even if the specific mechanisms differ across diseases.

Comparisons with other immune-mediated CNS disorders further illustrate these distinctions. In neuromyelitis optica spectrum disorder (NMOSD) and myelin oligodendrocyte glycoprotein antibody-associated disease (MOGAD), disease mechanisms are more directly linked to defined autoantibodies and inflammatory attacks, with a closer correspondence between immune activity and clinical worsening (84–86). By contrast, MS is characterized by a more complex and only partially coupled relationship between inflammation, tissue injury, and long-term progression. These differences do not place MS outside the spectrum of immune-mediated disease but highlight variation in causal structure and temporal organization across CNS inflammatory disorders.

MS therefore contributes less by providing generalizable answers than by clarifying the types of questions that autoimmune theory must address. It illustrates why unifying theoretical frameworks have proven difficult to formulate and why future progress may depend as much on conceptual clarity and theoretical coherence as on the identification of additional molecular mechanisms.

## Discussion

Over more than a century of investigation, MS has played a distinctive role in autoimmune disease research, not because of its prevalence or clinical burden alone, but because it has repeatedly exposed the strengths and limitations of prevailing autoimmune concepts. The central proposition developed in this article is that MS cannot be fully understood through a single linear autoimmune framework operating uniformly across all disease stages. Instead, our perspective suggests that disease susceptibility, inflammatory activity, and progressive neurological worsening represent mechanistically related but only partially coupled dimensions of disease, whose relative importance changes over time. The disease has served as a setting in which core assumptions about immune causation, specificity, and disease evolution could be tested against unusually rich pathological, experimental, genetic, and longitudinal clinical evidence. In doing so, it delineates where autoimmune theory provides explanatory power and where it requires refinement.

A central lesson from MS is that autoimmune theory has been most successful when applied to questions of disease initiation and early inflammatory activity. Immune-mediated mechanisms, antigen-directed responses, and genetic associations within immune pathways together form a coherent account of how disease can begin. At the same time, MS has consistently demonstrated that these mechanisms alone are insufficient to explain the full course of disease, particularly neurodegeneration and long-term progression. Rather than contradicting autoimmune theory, this defines the conditions under which it is explanatory and where additional conceptual layers are required.

MS also shows that autoimmune disease cannot be fully understood through the accumulation of mechanistic findings alone. Despite an ever-expanding catalog of molecular pathways, immune cell subsets, genetic loci, and environmental associations, core conceptual questions remain unresolved. These include how causal roles are distributed across different disease phases, how immune-mediated injury interacts with tissue-specific properties over time, and how risk, inflammatory activity, and gradual worsening relate to one another without collapsing into a single causal chain. The persistence of these questions underscores the need for explicit theory development alongside experimental research and the systematic analysis of clinical disease trajectories.

Looking forward, progress in autoimmune disease research is likely to depend on the refinement of integrative models that explicitly distinguish between mechanisms relevant to disease susceptibility, those governing inflammatory activity, and those shaping long-term tissue damage and repair. Such models must also incorporate temporal structure, acknowledging that the relative importance of immune activation, prior immune conditioning, tissue response, and repair capacity can change over the disease course. Equally important will be the development of biomarkers capable of capturing these different disease dimensions. In MS, quantitative MRI approaches and advanced image analysis may help capture diffuse tissue injury and neurodegeneration more directly than conventional imaging, although their broader clinical implementation remains challenging (87). Emerging computational approaches, including AI-assisted image analysis (88), may further improve detection of subtle structural changes, but their role in monitoring disease progression and informing treatment decisions remains to be established.

More broadly, the MS experience highlights why unifying theories of autoimmunity have remained elusive. Autoimmune diseases share immune involvement, but they differ in causal architecture, temporal organization, and the balance between immune-driven and tissue-driven processes. Rather than seeking a single explanatory framework applicable to all autoimmune conditions, future theoretical efforts may be better directed toward defining classes of causal structure, identifying shared principles while allowing for disease-specific variation.

The contribution of MS therefore lies not in offering a generalizable template but in sharpening the questions that autoimmune theory must address. By revealing where classical assumptions hold and where they break down, MS points toward the need for greater conceptual clarity and theoretical integration. Advancing autoimmune research will depend on how existing evidence is organized, interpreted, and integrated into coherent explanatory frameworks.

## Data Availability

The original contributions presented in the study are included in the article/supplementary material. Further inquiries can be directed to the corresponding author.
